# Supplementary material for: Modelling Identity Disturbance: A Network Analysis of the Personality Structure Questionnaire (PSQ)
Source: Int J Environ Res Public Health. 2022 Oct 24;19(21):13793. doi: 10.3390/ijerph192113793 (PMC9656866; doi:10.3390/ijerph192113793)
Supplement: Supplementary file 1 [file ijerph-19-13793-s001.zip › ijerph-1934043-supplementary.pdf]

Supplementary Materials

This supplementary document contains:

1. Subsample network and centrality comparisons:
    a. S1: Adult versus adolescent subsamples
    b. S2: Clinical versus community subsamples
    c. S3: Complex mental health versus common mental health problem subsamples

Subsample network comparisons

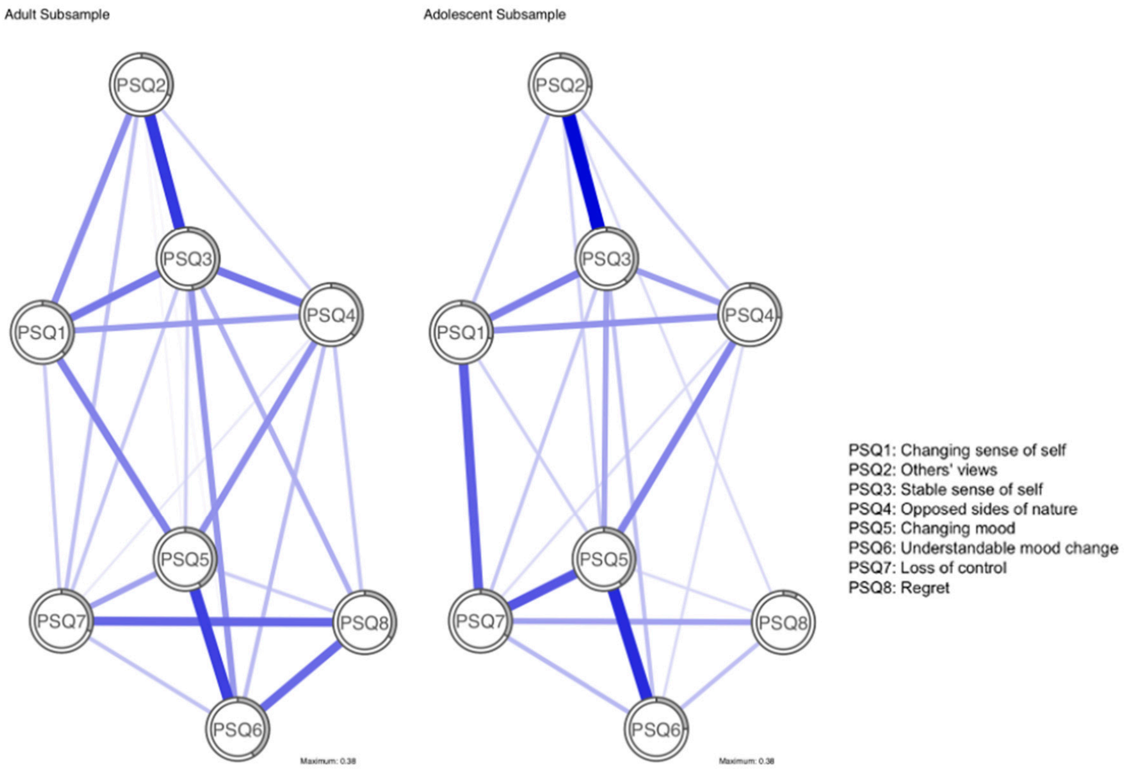

(**a**)

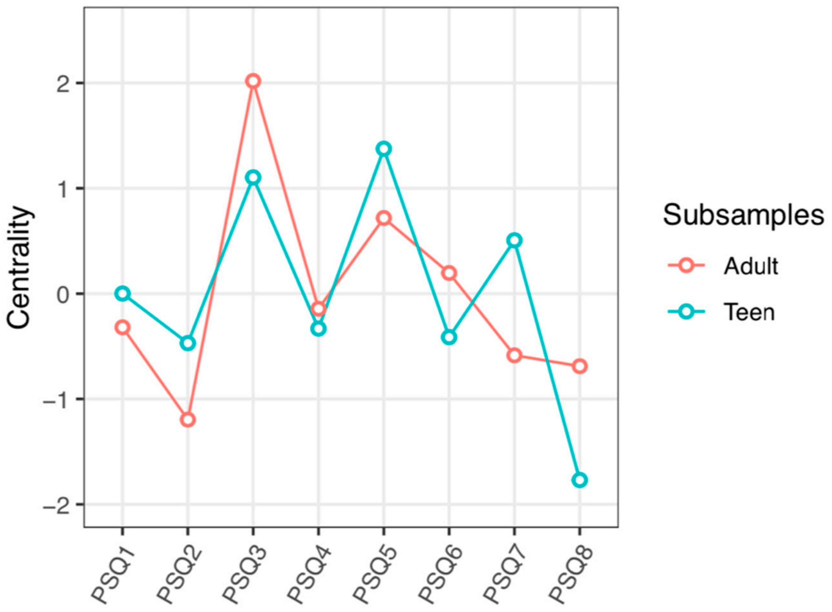

(**b**)

Figure S1. (a) Jointly estimated networks of identity disturbance in adult and adolescent subsamples (from Italian sample only). Positive edges are represented by blue lines and negative edges by red lines. Shaded areas surrounding nodes represent node predictability. (b) Plot of node strength centrality comparison between age subsamples.

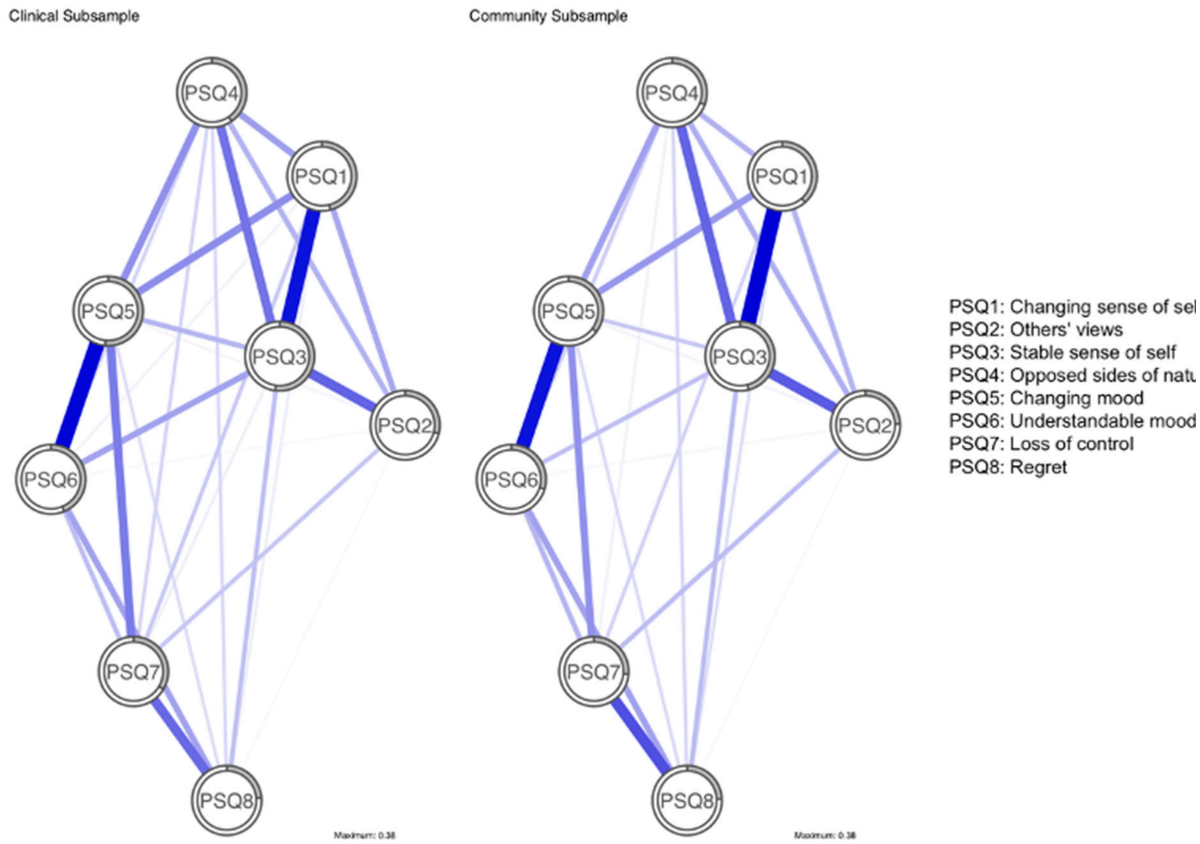

(**a**)

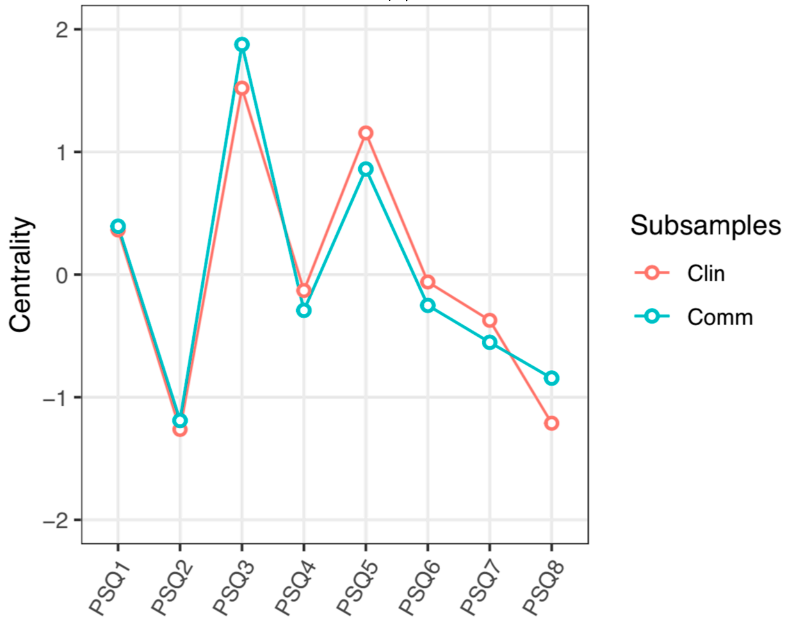

(**b**)

Figure S2. (a) Jointly estimated networks of identity disturbance in clinical and community subsamples. Positive edges are represented by blue lines and negative edges by red lines. Shaded areas surrounding nodes represent node predictability. (b) Plot of node strength centrality comparison between recruitment subsamples.

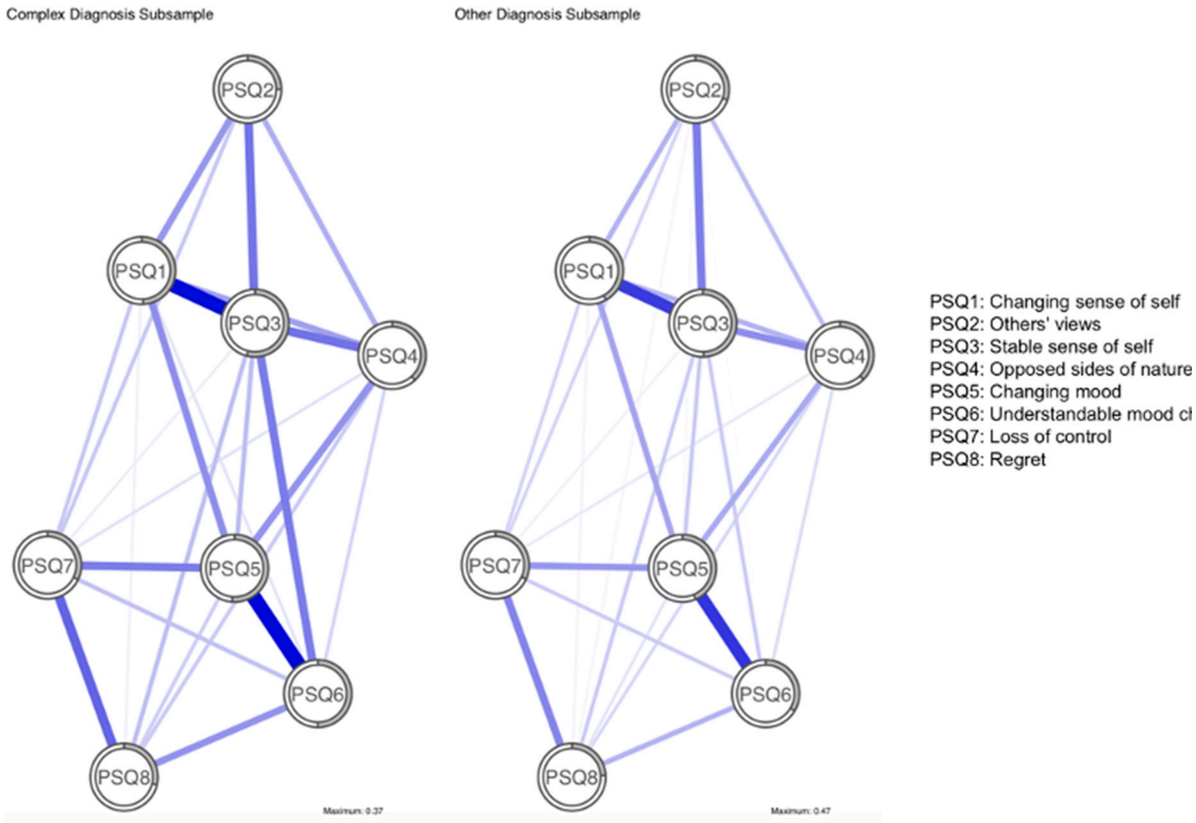

(**a**)

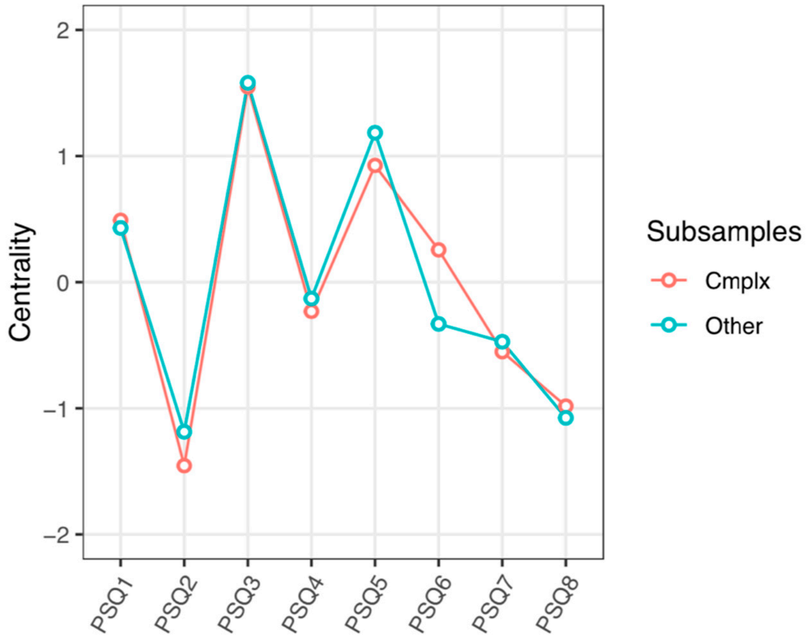

(**b**)

Figure S3. (a) Jointly estimated networks of identity disturbance in complex and common mental health problems subsamples. Positive edges are represented by blue lines and negative edges by red lines. Shaded areas surrounding nodes represent node predictability. (b) Plot of node strength centrality comparison between diagnosis subsamples.
